# Molecular Basis of Epstein–Barr Virus Latency Establishment and Lytic Reactivation

**DOI:** 10.3390/v13122344

**Published:** 2021-11-23

**Authors:** Takayuki Murata, Atsuko Sugimoto, Tomoki Inagaki, Yusuke Yanagi, Takahiro Watanabe, Yoshitaka Sato, Hiroshi Kimura

**Affiliations:** 1 Department of Virology and Parasitology, Fujita Health University School of Medicine, Toyoake 470-1192, Japan; asugi@fujita-hu.ac.jp; 2 Department of Virology, Nagoya University Graduate School of Medicine, Nagoya 466-8550, Japan; tinagaki@ucdavis.edu (T.I.); yusuke.yanagi@med.nagoya-u.ac.jp (Y.Y.); t.nabe.watanabe@med.nagoya-u.ac.jp (T.W.); yssato@med.nagoya-u.ac.jp (Y.S.); hkimura@med.nagoya-u.ac.jp (H.K.); 3 Clinical Research Center, National Hospital Organization Nagoya Medical Center, Nagoya 460-0001, Japan; 4 Department of Dermatology, School of Medicine, University of California Davis (UC Davis), Sacramento, CA 95817, USA

**Keywords:** EBV, latency, reactivation, transcription, epigenetics, oncogenesis

## Abstract

Epstein–Barr virus (EBV) is a causative agent of infectious mononucleosis and several types of cancer. Like other herpesviruses, it establishes an asymptomatic, life-long latent infection, with occasional reactivation and shedding of progeny viruses. During latency, EBV expresses a small number of viral genes, and exists as an episome in the host–cell nucleus. Expression patterns of latency genes are dependent on the cell type, time after infection, and milieu of the cell (e.g., germinal center or peripheral blood). Upon lytic induction, expression of the viral immediate-early genes, BZLF1 and BRLF1, are induced, followed by early gene expression, viral DNA replication, late gene expression, and maturation and egress of progeny virions. Furthermore, EBV reactivation involves more than just progeny production. The EBV life cycle is regulated by signal transduction, transcription factors, promoter sequences, epigenetics, and the 3D structure of the genome. In this article, the molecular basis of EBV latency establishment and reactivation is summarized.

## 1. Introduction

Epstein–Barr virus (EBV) is an enveloped virus with virions 100–200 nm in diameter [1]. A linear, double-stranded viral DNA genome is contained in the icosahedral nucleocapsid, and the component between the envelope and nucleocapsid is termed the tegument. The EBV genome is about 175 kb and encodes about 80 open reading frames and 44 non-coding RNAs. It is a member of the γ-herpesvirus subfamily, together with Kaposi sarcoma-associated herpesvirus (KSHV).

EBV was discovered in 1964 in an endemic Burkitt lymphoma (BL) cell culture [1]. As the fourth herpesvirus to be identified, it was named human herpesvirus 4 (HHV-4). Although discovered in an African tumor specimen, it was soon clear that EBV is ubiquitous, irrespective of race, nationality, sex, hygienic status, occupation, and presence of tumor. Indeed, more than 95% of adults worldwide are asymptomatically infected with EBV. The spread of EBV is mediated by saliva. Many people are infected with the virus during childhood, i.e., as babies or toddlers, mostly via family members. Others are infected in their teens or later, usually through friends or partners. Infection during infancy is not associated with obvious symptoms, whereas infection during/after the teenage years frequently causes infectious mononucleosis (IM) [2]. After initial infection, the virus establishes latency predominantly in B cells and cannot be eradicated. From time to time, latent EBV is reactivated, executes its lytic cycle, and produces progeny viruses, which are excreted in saliva [3,4]. Less frequently, persistent EBV infection results in the development of several types of cancers in lymphocytes and epithelial cells. Such EBV-associated cancers include BL, Hodgkin’s lymphoma (HL), post-transplant lymphoproliferative disorder (PTLD), NK/T cell lymphoma (NKTCL), chronic active EBV infection (CAEBV), diffuse large B cell lymphoma (DLBCL), nasopharyngeal carcinoma (NPC), and gastric carcinoma (GC) [5].

Herpesviruses establish latency, although the site of latency depends on the virus (Table 1) [6]. For example, herpes simplex virus 1 (HSV-1) lurks in neurons and human cytomegalovirus (HCMV) in precursor cells of myeloid lineages. Two human γ-herpesviruses, EBV and KSHV, maintain latency predominantly in B cells in vivo. It must also be mentioned that latent infection of EBV is more complex and variable than those of other herpesviruses. The latent form of EBV infection is further categorized into five patterns, Latency 0 to III (Table 2), depending on the viral latent gene expression [7]. The molecular basis of switching to the lytic cycle (i.e., lytic switch genes, transcriptional activators, repressors, and signalings) also differs among herpesviruses, likely because the site of latency is different (Table 1). In this review article, the molecular mechanisms of EBV latency and reactivation are explained.

## 2. Establishment of Latency

**Figure 1:**
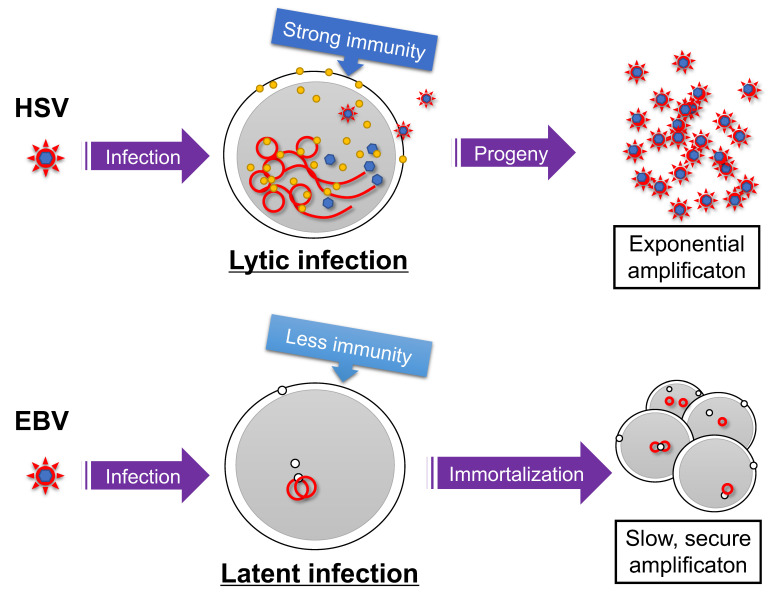
Survival strategies of herpesviruses. Infection of herpes simplex virus (HSV) causes lytic infection and the production of many progeny viruses, although the infected cells and virions may be targeted by the host immune reaction. Latent infection is maintained when the Epstein–Barr virus (EBV) infects a B cell, and the virus genome is amplified in synchronization with host cell amplification after immortalization. Red line indicates viral genome. Yellow and white circles represent viral lytic and latent gene products, respectively.

Because EBV infection of differentiated epithelial cells results in the lytic cycle [8,9], it is speculated that differentiated epithelial cells (likely stratified squamous epithelium in oral cavity or pharynx) are the initial site for EBV infection and multiplication in vivo. On the other hand, EBV infection of B cells in vitro predominantly causes latent infection, suggesting that B cells are the site for EBV latency. In addition, EBV chooses latent infection in many other cell types, such as established epithelial or T/NK cell lines [10,11]. Such latency-prone infection is a distinctive feature of EBV (and also KSHV); other herpesviruses (e.g., HSV) generally execute the lytic program upon de novo infection of many types of cells (Figure 1). This may be related to the fact that γ-herpesviruses have oncogenic potential. HSV must replicate as early as possible before being cleared by host immunity, because lytic replication cycle produces large quantities of viral antigens. By contrast, latent infection of EBV promotes the growth of infected B cells. Viral genomes are amplified in S phase, when cell proliferation is induced. Therefore, γ-herpesviruses use a survival strategy of slow replication in synchronization with host–cell genome replication. Mechanistically, such latency-prone infection of EBV upon de novo infection can be explained by a viral unique transactivator BZLF1 and epigenetics [12]. EBV genome DNA, released from a nucleocapsid, lacks CpG methylation and therefore BZLF1 cannot immediately bind and activate viral lytic promoters, as further detailed later.

**Figure 2:**
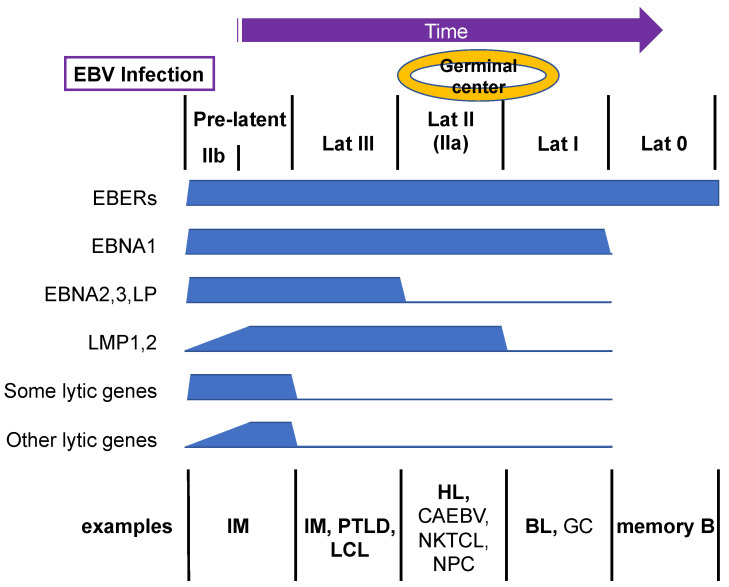
Transition of EBV gene expression upon primary B-cell infection. EBV infection of primary human B cells triggers the expression of both latent and lytic viral genes, which is termed the pre-latent phase. Subsequently, viral genes are silenced gradually. Example representative cells with the indicated patterns of viral gene expression in vivo are shown. Bold letters are B cells, and non-bold letters indicate non-B cells.

Although EBV predominantly establishes latent infection in primary B cells, closer observations have revealed that lytic genes are also expressed in addition to latent genes for several weeks upon de novo infection, yet productive viral DNA synthesis does not take place [11,13]. This period is called the pre-latent abortive lytic phase or, more simply, the pre-latent phase (Figure 2). The reason for lytic gene expression before establishment of latency is unclear, but it is possible that leaky expression of lytic genes during the pre-latent phase is beneficial for immortalization or evasion of cell death [14]. For example, EBV lytic genes include two anti-apoptotic BCL2 homolog genes (BALF1 and BHRF1) [15] and genes that downregulate human leukocyte antigen (HLA) gene expression (e.g., BNLF2A, BGLF5, BILF1, and BDLF3) [16]. The molecular mechanism of this pre-latent lytic gene expression is still elusive, but it may simply be accounted for by uncontrolled gene expression from the EBV genome, which lacks suppressive epigenetic modifications, such as CpG methylation. It may also be explained, at least partly, by the viral mRNAs incorporated into the virion [17], which can be released into cells upon infection.

Immediately after infection of primary B cells, the expression of six EBV nuclear antigen (EBNA) genes, EBNA1, 2, 3A, 3B, 3C, and LP, peaks, and the high levels are maintained for a long period [18,19]. The nature and functions of these EBV latent genes have been reviewed elsewhere [20]. A cluster of W promoters (Wp) is activated to induce EBNAs upon infection. Each Wp is mapped to each component of internal repeat 1 (IR1), and thus Wp is present as multiple copies. In fact, Wp is vital for EBV because it is responsible for the expression of EBNA2, a transcriptional cofactor and master regulator of the reprogramming of the resting B-cell transcriptome (e.g., temporal induction of MYC) [21]. The Wp exhibits maximum activity in B cells [22], which is mediated by the B-cell-specific transcription factor PAX5 and CREB/ATF [23,24,25]. The high number of Wp (usually about 10 copies) enables high EBNA2 expression immediately after the entry of EBV into B cells [26]. EBNA2 expression causes repression of Wp and activation of the C promoter (Cp) [27]. The physiological role of this switching of promoter usage is not understood, because the Cp is not required for B-cell growth transformation [28]. However, the Cp may be necessary in vivo. The Cp is located upstream of the Wp as a single copy and shows maximal activity in B cells [22]; this activity is mediated by EBNA2 and other factors, including OCT2, E2F, ARID3A, NF-Y, and SP1 [29,30].

Latent membrane proteins (LMPs), such as LMP1, 2A, and 2B, are expressed at low levels during the first few days after B-cell infection (Figure 2) [19,31]. The mechanism of initial suppression of LMP1 was long elusive [32], but MYC, which is induced immediately after B-cell infection by EBNA2, is responsible for LMP1 suppression [33]. Subsequently, the expression of LMPs gradually increases over a few weeks. This increase is mediated in part by EBNA2 [34,35], although other transcription factors, such as nuclear factor-κB (NF-κB), EBF1, PU.1, IRF7, and CREB/ATF, may also be involved [36,37,38,39,40]. Expression of LMP1 is important because LMP1 is a constitutively active oncogene of EBV. It mimics CD40 and elicits multiple signals, such as NF-κB and mitogen-activated protein kinase (MAPK), needed for cell growth and survival [41].

There seem to be two main expression patterns of lytic genes. The expression of several lytic genes, such as BHRF1, BOLF1, and BPLF1, is induced in high amounts immediately after infection, and those levels are maintained for weeks, similar to EBNAs, whereas the expression of other lytic genes increases gradually, reminiscent of LMPs (Figure 2) [19,42]. The reason for the different expression patterns of lytic genes is not clear, but it is possible that swiftly induced lytic genes are influenced by EBNA genes, given that they are frequently adjacent to each other. Thus, the first few days after EBV infection are characterized by low expression of LMPs and lytic genes, whereas EBNA genes are abundantly expressed, a pattern termed latency IIb (Figure 2) [31]. This pattern of EBV infection is observed in some B cells of IM patients. The pre-latent phase continues for weeks (or months) until the expression of lytic genes is silenced, and the virus establishes latency III, where latent genes are fully expressed and lytic genes are suppressed. Latency III cells can be seen in the presence of weak host–cell immunity, as seen in IM, PTLD, and the lymphoblastoid cell line (LCL) (Figure 2). LCL is a B cell line immortalized by infection of EBV.

## 3. Restriction of Latent Genes

If naïve B cells are infected with EBV, they move to germinal centers of the lymph nodes and spleen, where expression of EBNA2, EBNA3, and LP is suppressed to establish latency II (or IIa) (Figure 2). EBNA1–3 and LP are transcribed from the Cp in latency III, but the promoter is silenced and EBNA1 is instead expressed from the Qp in latency II. Because Cp activation is predominantly dependent on EBNA2 [43], the Cp is downregulated in association with decreased EBNA2. However, the trigger for decreased EBNA2 in germinal centers is unknown. Germinal center-specific transcriptional repressors, such as BCL6, may play a role in this process, because EBNA2 and BCL6 expression is inversely correlated [44]. Furthermore, treatment of latency III cells with IL-21 resulted in decreased Cp activity [45]. Because IL-21 induced BLIMP1 and OCT2, these factors may be involved in repression of the Cp. Although EBNA2, EBNA3, and LP are suppressed, LMP1, 2A, and 2B are expressed in latency II even in the absence of EBNA2. Cytokines, including IL-4, IL-6, IL-10, IL-13, and IL-21, have been implicated in EBNA2-independent expression of LMP1 [45,46,47,48,49], and the transcription factors NF-κB, AP-2, IRF7, C/EBP, and STAT in LMP1 promoter activation [37,38,39,46,47,50,51]. The HL, derived from B cells, typically shows this expression pattern. Some non-B-cell cancers, such as CAEBV, NKTCL, and NPC, also use this mode—Wp/Cp is not active in cells other than B lymphocytes.

LMP1, LMP2A, and LMP2B can be silenced in BL and GC, which is termed latency I (Figure 2) [11]. The mechanism of this silencing is unclear, but because latency III cell lines can be isolated from latency I parental cell lines in cell culture, which lacks immune pressure, elimination of cells that express LMPs by host immunity likely plays a role in the process.

The mostly silenced mode is latency 0, in which only EBERs are expressed, in peripheral memory B cells (Figure 2) [11]. The mechanism of this silencing of EBNA1 is not known, but host immunity may be involved.

## 4. Reactivation from Latency

**Figure 3:**
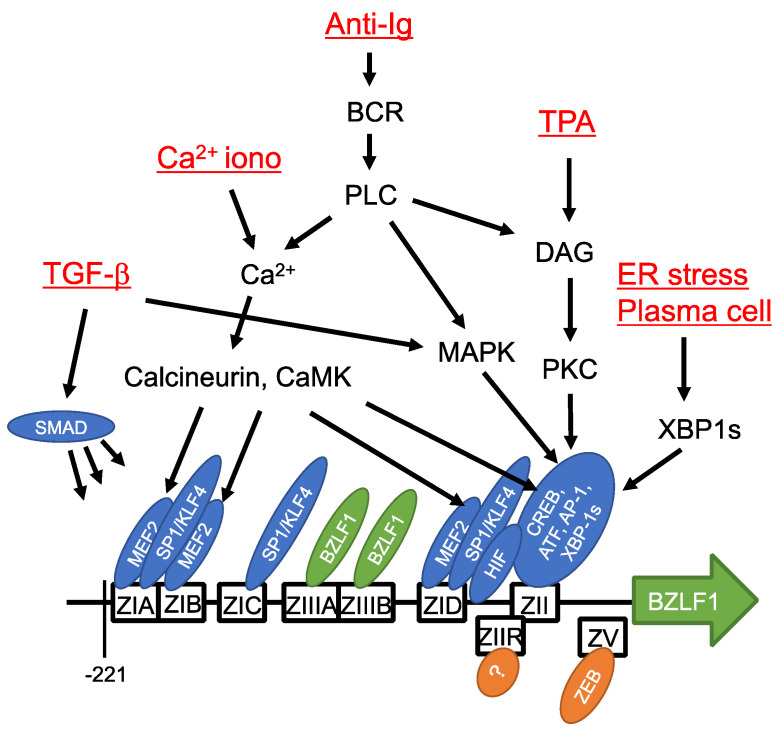
*Cis*- and *trans*-acting elements of BZLF1 promoter and signaling pathways. *Cis*-acting elements of the BZLF1 promoter sequence sufficient for induction in response to lytic stimuli are depicted as boxes. Transcription factors that bind the elements are indicated by ellipses. Inducing reagents/stimuli and signaling molecules are also shown. Modified from [54].

Reactivation of EBV can be induced in cultured cells by chemical reagents, such as phorbol ester, calcium ionophore, histone deacetylase (HDAC) inhibitors, and DNA methylase inhibitors, and by biological stimulation through transforming growth factor β (TGF-β), anti-immunoglobulin, hypoxia, reactive oxygen species, and temperature shifts (Figure 3) [52,53,54]. Although the trigger is unknown, EBV reactivation can occur in response to a variety of stimuli (Figure 3).

EBV encodes two immediate-early (IE) genes, BZLF1 (also known as Z, Zta, ZEBRA, or EB1) and BRLF1 (R or Rta) [55]. Expression of the IE genes is suppressed in the latent phase but accelerated by inducing reagents or stimulation. BZLF1 and BRLF1 are tandemly and adjoiningly mapped in the EBV genome near the EBNA1 gene. The fact that these IE genes are situated within the intron sequence of EBNA1 in a complementary orientation is interesting, because HSV LAT is complementary to ICP0.

BZLF1 is a viral transcription factor, similar to host AP-1 and CREB, and has a basic leucine zipper (bZip) motif [56,57]. It forms a homodimer via the bZip motif, binds to DNA and induces transcription of viral downstream genes [58]. BZLF1 binds to binding motif similar to AP-1, called BZLF1-responsive element (ZRE), which are abundant in the promoters of viral lytic genes, especially early genes, and induces transcription [59,60]. Uniquely, this transcription factor binds to CpG-methylated DNA [61,62,63]. This enables BZLF1 to activate transcription of the viral lytic genes silenced by CpG methylation in latent cells. In addition, BZLF1 functions as an origin of lytic replication (ori-Lyt) binding protein in lytic viral DNA synthesis [64]. By contrast, BRLF1 is a transcriptional activator that acts in three ways [55]. First, it binds directly to BRLF1-responsive element (RRE) of promoter and activates transcription as a transcription factor [65,66]. Second, it is a transcriptional co-factor that binds indirectly to GC-rich motifs by binding to other cellular transcription factors, such as SP1 and OCT-1 [67,68]. Third, it regulates signaling pathways, such as MAPK and phosphatidylinositol 3-kinase (PI3K) [69,70]. Both IE genes are essential for lytic reactivation, because the lytic cycle is blocked by knockout of either gene. Furthermore, exogenous overexpression of BZLF1 or BRLF1 in latently infected cells can trigger lytic reactivation. BRLF1, but not BZLF1, is needed for EBV reactivation in EBV-positive normal oral keratinocytes [8]. However, BZLF1 is presumed to be more important because more efficient reactivation can be achieved by exogenous expression of BZLF1 than of BRLF1, at least in many cell lines.

Expression of BZLF1 is regulated at the transcriptional level. Therefore, research has focused on the BZLF1 promoter sequence (Figure 3). Reporter assays showed that a short sequence in the BZLF1 promoter (−221 to +12) was sufficient for its activation by 12-*O*-tetradecanoylphorbol-13-acetate (TPA) [71] and anti-immunoglobulin [72] (Figure 3). This short promoter sequence consists of four copies of ZI, and one ZII and two ZIII motifs. ZIA, B, and D are bound by MEF2 family transcription factors and ZIA, C, and D are bound by SP1/KLF4 [73,74,75]. MEF2 is a negative regulator bound to the promoter in latency; it activates the promoter in response to calcium signaling. SP1/3 proteins are ubiquitous host transcription factors and their stable binding to ZIA, C, and D underscores the basal activity of the BZLF1 promoter [73]. KLF-family transcription factors have similar consensus binding motifs to SP1. KLF4 binds and activates the BZLF1 promoter [75] in a differentiation-dependent manner [76]. The sequence of the ZII domain is similar to the consensus binding sequence of CREB and AP-1, and is bound by a range of bZip transcription factors [71,77,78] needed for promoter activation by various stimuli. XBP-1 protein is inactive unless activated by splicing upon endoplasmic reticulum (ER) stress, and the spliced form of XBP-1 (XBP-1s) is associated with plasma cell differentiation. XBP-1s binds to the ZII element and activates the BZLF1 promoter [77]. Once produced, BZLF1 protein, as a viral transcription factor, activates its own promoter by binding to the ZIIIA and B domains [79]. HIF-1 binds to hypoxia-response element (HRE) between ZID and ZIIR, triggering BZLF1 promoter activation [80]. The BZLF1 promoter is activated by TGF-β via MAPK signaling, as well as by binding of SMAD [81].

Two major *cis*-acting elements are crucial for negative regulation of the BZLF1 promoter (Figure 3). ZEB1/2 proteins bind to the ZV and ZV’ domains located around the transcriptional start site of BZLF1 and suppress transcription of BZLF1 [82,83]. The ZIIR element, located between ZID and ZII, is a strong silencer of the promoter [84,85], although the ZIIR-binding protein has not yet been identified. Other suppressive factors recently identified include MYC and KAP1 [86,87].

The BRLF1 promoter is activated by transcription factors, such as SP1, EGR1, BLIMP1, NF1, and BZLF1, and suppressed by YY1 and ZEB [61,88,89,90,91,92].

**Figure 4:**
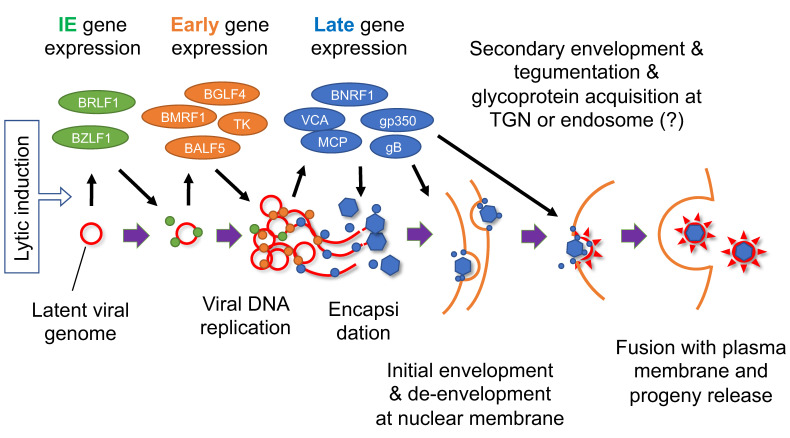
Schematic diagram of EBV lytic cycle. Lytic induction results in a coordinated cascade of viral gene expression and viral DNA replication, followed by encapsidation, envelopment, and progeny production. Red line indicates viral genome. Green, orange, and blue circles represent viral IE, early, and late gene products, respectively. TGN, *trans*-Golgi network. Modified from [111].

When IE genes are expressed at a certain level, these transcriptional activators induce transcription of viral early genes (Figure 4). Among the early class genes, BMLF1/BSLF2 (also named SM, Mta, and EB2), the homolog of HSV ICP27, encodes an essential protein involved in viral gene expression by affecting multiple processes, including RNA metabolism, transcription, and translation [93,94,95,96,97,98,99,100]. Other early gene products include enzymes involved in nucleotide metabolism, such as thymidine kinase (TK) and ribonucleotide reductase (RR), which presumably play supportive roles, especially in resting cells (Table 3). In addition to six essential viral replication genes (BALF5, BMRF1, BALF2, BBLF4, BSLF1, BBLF2/3), BKRF3 (uracilDNA glycosylase) and BZLF1 (oriLyt-binding protein) have also been identified to be crucial for replication [64,101,102,103] (Table 3). These viral factors trigger viral DNA replication in the replication compartment of the nucleus [64]. After viral DNA replication, a complex of viral gene products, dubbed the viral preinitiation complex (vPIC), mediates late gene expression from the amplified viral DNA [104,105,106,107,108,109,110] (Table 4). Basically, this complex recognizes two features of DNA; TATT motif instead of TATA at the promoter, and lack of epigenetic modifications, such as methylation. The TATT motif is specifically found in the late gene promoters and one of the vPIC, BcRF1, recognizes the motif. Lack of epigenetic marks is an earmark of newly synthesized viral DNA.

Late genes include many viral structural genes, such as major capsid protein (MCP), viral capsid antigen (VCA), major tegument protein (BNRF1), and glycoproteins (gB and gp350). Encapsidation takes place in the nucleus, and the nucleocapsid is transported to the cytoplasm through the nuclear membrane, a process in which two viral proteins, BFRF1 and BFLF2, and other viral and cellular factors are involved [112]. During this nuclear egress, the nucleocapsid must twice cross the lipid bilayer membrane. The nucleocapsid buds into the inner nuclear membrane to obtain an envelope (primary or initial envelopment), which is removed by fusion with the outer nuclear membrane. The naked nucleocapsid next buds into a membranous organelle in the cytoplasm (possibly the *trans*-Golgi network (TGN), endosome, or a related organelle). Tegument components are also incorporated at this step of secondary envelopment. Finally, fusion of the organelle with the plasma membrane releases mature progeny virions. The functions of EBV lytic genes are summarized elsewhere [113].

## 5. Epigenetic Regulation of Latency Establishment

**Figure 5:**
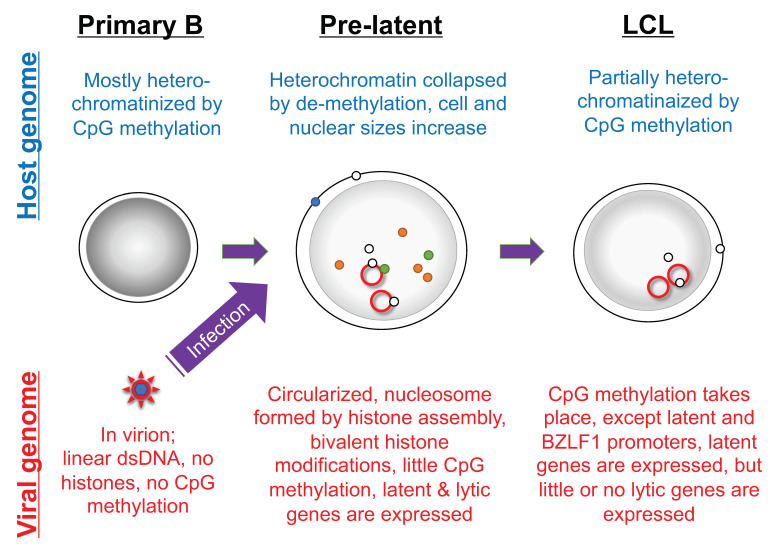
Morphology and epigenetic state of primary B cells infected with EBV. The EBV genome in virions is devoid of nucleosome structure and CpG DNA methylation, but is modified by histones and CpG methylation in infected cells. The host cell morphology and genome are significantly influenced by EBV infection. The red line indicates latent viral genome. Dark gray shadows in nuclei indicate strong heterochromatinization. Green, orange, and blue circles represent viral lytic gene products, and white circles represent viral latent gene products.

The episomal EBV genome in latently infected cells is modified by CpG methylation and assembled with histones to form a chromatin structure. However, EBV genome DNA in the viral nucleocapsid is devoid of epigenetic marks, such as histones and CpG methylation (Figure 5) [114,115,116]. When primary B cells are infected with EBV, the EBV genome is first chromatinized by histone assembly, followed by histone modification, for example of histone H3 K27 trimethylation (H3K27me3) and H3K9me3 [117]. Those suppressive histone modifications restrict EBV gene expression but permit leaky expression of many viral genes, which is recognized as lytic gene expression in the pre-latent phase. Suppressive histone modifications, such as H3K27me3, H4K20me3 and H3K9me3, during the pre-latent phase are not sufficient for complete suppression of viral genes, likely because the viral genome is associated with activating histone modifications, such as histone H3K4me3 and acetylation (Figure 5). Interestingly, the major tegument protein BNRF1 associates with histone chaperone molecules upon infection of primary B cells and increases the active histone mark, H3K4me3, on the viral genome [118]. In addition to those histone lysine methylations, a series of reports have indicated that arginine methyltransferases, especially PRMT5, play an important role in transformation [119,120,121,122]. After histone modifications, CpG methylation of viral DNA occurs, over weeks to months [116]. After completion of CpG methylation, viral lytic gene expression is strongly suppressed (Figure 5).

In contrast to viral genome chromatinization, suppressive histone markers and CpG methylation of the host genome are disrupted when primary B cells are infected, and gradually restored thereafter [123,124,125]. However, even in established LCL, CpG methylation of the host genome is significantly lower compared to that in primary B cells (Figure 5). It is speculated that immediate collapse of host genome CpG methylation upon infection is needed for overexpression of the key host genes for growth transformation.

The situation is different in epithelial cells. Viral genomic DNA starts to be CpG-methylated as early as day 11 and is completed by day 17 after infection of an immortalized normal gastric epithelial cell line. Demethylation of the host genome is not detected in the epithelial cell line.

Instead, CpG methylation takes place from day 17 to 28, a period associated with decreased expression of genes involved in tumor suppression and cell differentiation [126]. It is assumed that, in epithelial cells, suppressive histone modifications also take place before CpG DNA methylation, as in B cells [127]. The reason why large-scale host genome CpG demethylation does not occur in epithelial cells is unclear, but differences in the cell cycle may account for delayed CpG methylation of EBV genome DNA in B lymphocytes. Primary B cells used in such experiments are in G_0_ phase and thus completely growth-halted, whereas the epithelial cells used are established cell lines or immortalized normal epithelial cell lines. In resting cells, gene expression is generally suppressed to a minimal level by CpG methylation, but immortalized cells must express genes that support proliferation.

EBV super-enhancers are clusters of enhancers dense with transcription factors and cofactors, and mediate strong activation of specific genes that regulate cell identity and fate [128]. EBV super-enhancers are typically composed of EBNA2, EBNA-LP, EBNA3A, EBNA3C, NF-κB, and RBPJκ, and are associated with a high level of histone acetylation [129,130]. Chromatin loop structures are formed by CTCF and assembling enhancers, with which RNAPII is frequently associated. Induction of critical host genes, such as MYC, BCL2, IRF4, MCL1, EBF, and RUNX3, in primary B-cell infection is dependent on EBV super-enhancers [129,130,131,132].

## 6. Epigenetic Regulation of Latent Gene Restriction

Silencing of viral genes in infected cells by histone modification and CpG methylation is not a complicated concept. However, the maintenance of latency must be intricately regulated, because only a handful of appropriate latent genes should be expressed efficiently while other latent and lytic genes must be silenced. Excessive transcription of improper genes in an inappropriate context, or insufficient expression of necessary genes, would negatively affect the infected cells and virus genome. Moreover, the density of genes in the EBV genome is high (about 80 genes in 175 kb; ~2.2 kb per gene), in comparison to the human genome (about 20,000 genes in 3,000,000 kb; ~150 kb per gene). In this respect, CpG methylation of particular promoters plays a primary role. For example, Wp/Cp/Qp activity is inversely correlated with CpG methylation level of the promoter [114,133,134,135,136,137,138,139,140]. The LMP1 promoter is also regulated by CpG methylation [114,135,141,142]. TET2, which mediates the conversion of methylated CpG into hydroxymethylcytosine, ultimately leading to CpG demethylation, increases the expression of EBNAs and LMPs [143,144]. Furthermore, a component of polycomb repressive complex 1 (PRC1) and DNA methyltransferase (DNMT) enzymes are crucial for the suppression of EBNAs and LMPs [145]. These reports demonstrate the importance of CpG methylation in fine-tuning viral latent gene expression.

Heterochromatin tends to expand the suppressive region to neighboring euchromatin sequences. Therefore, once a part of the viral genome has been silenced by CpG methylation, the methylation may spread to neighboring promoters, which could be needed for maintenance of EBV latency. To block the ripple of suppression, however, EBV uses a host insulator protein, CTCF [146,147]. Qp must be kept active in latency I and II to mediate the expression of EBNA1 when Wp/Cp and other lytic promoters are silenced by CpG methylation. CTCF protein binds upstream of Qp and prevents suppressive epigenetic modifications, such as histone H3K9me3 and CpG methylation, thereby sustaining high promoter activity [148]. Moreover, analysis of the 3D organization of latent viral genome revealed that CTCF binding does not act as a simple epigenetic insulator. In complex with the host factor Cohesion, CTCF mediates loop formation of the viral genome in infected cells, which influences the epigenetic state and thereby alters gene expression. In latency I, CTCF/Cohesion mediates chromatin-loop formation between the origin of plasmid replication (OriP) and Qp, and increases Qp activity possibly via OriP-bound factors, EBNA1, and chromatin-remodeling factors, such as ORC and SNF2h [149]. In latency III, OriP forms a chromatin loop with the Cp and LMP1 promoters via CTCF/Cohesion, and EBNA1 and chromatin remodeling factors at the OriP mediate activation of Cp and the LMP1 promoter [149,150,151].

The silencing of latent genes is associated with higher levels of suppressive histone modification, such as histone H3K9me3 and H3K27me3 [152,153]. Appropriate transcriptional activation of latent gene promoters, including Cp/Wp/Qp and LMP promoters, is mediated by active histone marks, such as histone acetylation and H3K4me3 [151,153].

## 7. Epigenetic Regulation of Reactivation

Although almost all EBV lytic genes are silenced by CpG methylation in EBV-positive cell lines, CpG DNA methylation of the BZLF1 promoter is maintained at a low level, like those of latent promoters such as the Qp and LMP1 promoters [63,75,114]. Few CpG dinucleotides are present in the BZLF1 promoter, whereas CpG dinucleotides are denser in other latent and lytic promoters, including the BRLF1 promoter [63,75]. Therefore, it is assumed that BZLF1 transcription is not primarily silenced by CpG methylation in cell culture. Alternatively, the BZLF1 promoter is silenced by suppressive histone modifications, such as H3K27me3 and H3K9me3 [63,154,155,156]. Silencing of the BZLF1 promoter by histone methylation rather than CpG methylation is essential for prompt induction in response to lytic stimuli. These suppressive histone methylations are rapidly demethylated, but methyl-CpG is a stable mark that requires multistep demethylation (hydroxymethylcytosine formation, followed by glycosylase reaction and base excision repair, or de novo DNA synthesis) [157]. Moreover, the BZLF1 promoter is modified not only by suppressive, but also by activating, histone modifications [155,156], generating bivalent chromatin [158]. Therefore, the BZLF1 promoter is capable of activating the transcription of BZLF1 at any time. BZLF1 protein binds CpG-methylated DNA motifs and induces transcription of downstream viral genes [61,62,63]. These unique properties make BZLF1 a key factor for EBV reactivation from latency.

CpG DNA methylation of the viral genome is eliminated by de novo viral genome replication [114]. vPIC mediates viral late gene transcription only from unmethylated DNA, which is why late gene expression occurs after viral DNA synthesis [159].

## 8. Involvement of Lytic Genes in Oncogenesis

It was previously speculated that only latent genes (not lytic genes) were involved in the oncogenesis of EBV-associated cancers, because lytic genes were not readily detectable in those cancers. However, it is now understood that lytic genes are also expressed to some extent in cancer cells. Lytic genes of EBV are implicated in oncogenesis [14,160,161,162,163,164]. For example, in cell culture, pre-latent lytic gene expression contributes to B-cell transformation [116,165]. EBV in NPC has been shown to frequently express lytic genes and such lytic genes play an important role in oncogenesis [164]. EBV in CAEBV and other lymphomas, such as NKTCL and DLBCL, frequently has intragenic deletions, which presumably caused more lytic gene expression and tumor formation [163,166,167]. When humanized mice are infected with BZLF1-kockout EBV, tumors develop less frequently than in wild-type control EBV [168]. Knockout of BZL1 results in restriction of BZLF1 and other downstream lytic genes; thus, these lytic genes likely contribute to tumor development. By contrast, mutation of two repressive *cis*-acting elements, ZIIR and ZV, results in increased expression of BZLF1 and other lytic genes without viral DNA synthesis, and increased lymphoma formation [169].

Clinically oriented approaches have also been adopted. Two independent sequence analyses identified a nucleotide variation in the BZLF1 promoter region of EBV in NPC and lymphoma specimens [170,171], and this variant accumulated in BL and gastric carcinoma [172]. Introduction of this variation increased lytic gene expression [172]. The EBV genome in lymphoma specimens frequently bears an intragenic deletion in the BART region [167,173,174], the deletion of which upregulated lytic gene expression and promoted tumor formation in a humanized mouse model [175]. Essential lytic genes, such as viral DNA polymerase BALF5, are deleted in EBV-positive lymphomas, promoting a lytic phenotype and lymphomagenesis in mice [167].

Although the molecular mechanism by which the EBV lytic cycle contributes to oncogenesis is unknown, some lytic genes likely promote cancer formation. BCL2 homolog genes of EBV (BALF1 and BHRF1) protect infected cells from death [15]. BNLF2A, BGLF5, BILF1, and BDLF3 downregulate HLA and thereby protect infected cells from immunity [16]. BRLF1, BNRF1, BALF3, BGLF4, and BGLF5 have been implicated in genome instability [164]. These factors can confer advantages for infected cells. In addition, the proliferation of cells surrounding EBV-infected cells may be promoted. For example, production of the EBV homolog of IL-10, BCRF1, increases surrounding B-cell proliferation [176]. The lytic switch transcription factor BZLF1 enhances the production of cytokines, such as IL-6, IL-8, IL-10, IL-13, and VEGF [111], and increases the proliferation of surrounding cells.

## 9. Conclusions

Here, the life cycle of EBV and molecular basis thereof were summarized. EBV has established a complex life cycle that maximizes survival potential. Despite our immune system, humans cannot eliminate EBV from the body. To prevent EBV-associated disorders, which are not particularly rare, preventative and therapeutic measures are needed. Using its delicate life cycle of EBV against itself, disturbing viral gene expressions may provide possibility to interrupt EBV persistence and repress formation and maintenance of tumors. For example, our group has tried to suppress expression of viral oncogene LMP1 by HSP90 inhibitors to decrease CAEBV and NKTCL tumor growth [177,178]. Effects of epigenetic modifiers, such as HDAC inhibitors [179,180] and DNMT inhibitors [181,182], are also expected, especially for lytic induction therapy [183,184,185]. Further studies are needed, however, for such practical use.

## Figures and Tables

**Table 1:** Latency and lytic reactivation of human herpesviruses.

	Sub-Family	Site of Latency	Viral Gene Expressed in Latency	Trigger of Reactivation	Viral Lytic Swithch Gene	Transcriptional Activator of Reactivation	Transcriptional Repressor of Reactivation
HSV-1	a	neuron cells in trigeminal ganglia	LATs	stressors, immune suppression	ICP0, ICP4	HCF1, SP1, GR, KLF15, ROS	NGF
VZV		neuron cells in dorsal root ganglia	ORF63, VLT	stressors, immune suppression	IE62	HCF1, SP1, YY1, PI3K/AKT, JNK	
HCMV	b	myeloid lineages, hematopoietic progenitor cells, monocytes	UL138, UL81-82ast(LUNA), US28, UL144, UL111A(vIL10)	stressors, immune suppression, differentiation, growth factor	IE86(UL122), IE72(UL123)	PU.1, SP1, C/EBP, ATF/CREB, AP-1, NFkB, PPARg, RAR/RXR	CUX1/CDP, GFI-1, YY1, CTCF
EBV	g	B cells, epithelial cells	EBNAs, LMPs, EBERs	stressors, immune suppression, differentiation, groth factor, hypoxia	BZLF1(Zta), BRLF1(Rta)	SP1, KLF4, MEF2, C/EBP, ATF/CREB, AP-1, XBP1s, SMAD, HIF, ROS, P53, PI3K/AKT, TORC2, YAP/TAZ, TET2, ATM, BLIMP1, TAF-I/NAP1, NFATc1, CASP1	ZEB, NFkB, MYC, YY1, JDP2, NO, E2-2, JAK/STAT, SMUBP, OCT2, PAX5, BCL6, ARKL1, PARP1, CAF1/HIRA, KAP1
KSHV		B cells, endothelial cells	LANA, vCYC, vFLIP, Kaposins	stressors, immune suppression, differentiation, groth factor, hypoxia	K-Rta (ORF50)	C/EBP, AP-1, EGR1, XBP1, NFAT, HIF, ROS	NFkB, MYC, KAP1, PI3K/AKT, IFNs, IRFs, HES1, FOXO1

**Table 2:** EBV latency patterns.

Patterns	EBERs	EBNA1	LMP1/2	EBNA2/3/LP
Latency III	+	+	+	+
Latency IIa	+	+	+	
Latency IIb	+	+		+
Latency I	+	+		
Latency 0	+			

**Table 3:** Herpesviral genes involved in viral lytic DNA synthesis.

EBV	Necessity #	HSV	HCMV	Function
BZLF1 *	essential	UL9 *	UL84 *	oriLyt-binding
BALF5	essential	UL30	UL54	polymerase catalytic subunit (Pol)
BMRF1	essential	UL42	UL44	processivity subunit of polymerase
BALF2	essential	UL29	UL57	single-stranded DNA-binding protein (ssDNABP)
BBLF4	essential	UL5	UL105	helicase
BSLF1	essential	UL52	UL70	primase
BBLF2/3	essential	UL8	UL102	primase-binding protein
BKRF3	essential	UL29	UL114	uracil-DNA glycosylase, involved in DNA repair
BXLF1	supportive	UL23	–	thymidine kinase (TK)
BORF2	supportive	UL39	UL45	ribonucleotide reductase (RR) large subunit
BaRF1	supportive	UL40	–	ribonucleotide reductase (RR) small subunit
BLLF3	supportive	UL50	UL72	deoxyuridine triphosphate nuclotidohydrolase (dUTPase)

* These genes share the function, but have no sequential homology. # Necessity for EBV lytic replication.

**Table 4:** vPIC genes of EBV, KSHV, and HCMV.

EBV	HCMV	Presumed Function and/or Nature
BcRF1	UL87	TATT-binding protein, associates with RNAPII
BDLF3.5	UL91	
BDLF4	UL92	stabilized by phosphorylation
BFRF2	UL49	DNA-binding protein, having potential zinc-finger domain
BGLF3	UL95	hub of vPIC, phosphorylation increased association with BFRF2 and BVLF1
BVLF1	UL79	elongation factor (in HCMV)

## Data Availability

Not applicable.
